# A Case of Ticagrelor-Induced Sinus Pause

**DOI:** 10.7759/cureus.41821

**Published:** 2023-07-13

**Authors:** Nicholas T Pavlatos, Aangi Shah, Muhammad Khan, Vikash Jaiswal, Jishanth Mattumpuram

**Affiliations:** 1 Department of Internal Medicine, University of Louisville Hospital, Louisville, USA; 2 Department of Cardiology, University of Louisville Hospital, Louisville, USA; 3 Research and Academic Affairs, Larkin Community Hospital, Miami, USA

**Keywords:** continuous telemetry, myocardial ischemia and infarction, ekg abnormalities, adult cardiology, cardiac electrophysiology

## Abstract

Ticagrelor is an oral antiplatelet agent commonly used following percutaneous coronary intervention (PCI). There have been many reports describing bradyarrhythmias in the setting of ticagrelor use, most notably sinus pauses. This process is thought to be related to ticagrelor’s inhibition of human equilibrative nucleoside transporter (hENT1), which reduces cellular uptake of adenosine. We present a case of a 58-year-old male who experienced prolonged sinus pauses 38 hours after starting ticagrelor following ST-elevation myocardial infarction with subsequent PCI and stent placement.

## Introduction

Sinus pause or sinoatrial (SA) arrest is characterized by temporary cessation of sinus node activity, noted by the absence of both P waves and associated QRS/T complexes of at least three-second duration [1]. There are many cases in which sinus pauses occur in a non-diseased heart such as in well-conditioned athletes with increased vagal tone, the elderly population, and, most commonly, during sleep in the general population. Sinus pauses can also be the sequelae of other underlying disorders such as fibrosis of the SA node, myocardial ischemia, and metabolic abnormalities such as hypothermia or hyperkalemia [2,3].

Dual antiplatelet therapy (DAPT) combining aspirin with a P2Y12 inhibitor such as clopidogrel, prasugrel, or ticagrelor has become standard therapy after percutaneous coronary intervention (PCI) following acute coronary syndrome (ACS). Ticagrelor’s main mechanism of action is as a reversible direct antagonist of the P2Y12 adenosine 5’-diphosphate receptors, resulting in inhibition of platelet aggregation [4]. What is less well-known is that ticagrelor also inhibits human equilibrative nucleoside transporter (hENT1), which acts as an adenosine transporter in many different tissues. By blocking hENT1, ticagrelor inhibits cellular uptake of adenosine at clinically significant levels, potentially leading to SA and atrioventricular dysfunction. This phenomenon is most notable in the early hours after ACS where myocardial ischemia leads to increased levels of adenosine and has been shown to be significant in the first week following ticagrelor administration [5]. It should be noted that the active metabolites of clopidogrel and prasugrel do not have the same inhibitory properties on hENT1 and, therefore, do not share this same arrhythmogenic side effect [6,7].

## Case presentation

Our patient was a 58-year-old male with polysubstance use disorder and no known cardiac history who presented to the emergency department (ED) after a rollover motor vehicle accident. The patient was reportedly using heroin and lost consciousness while driving, causing him to run off the road and into the median. The patient was initially found to be unresponsive, received cardiopulmonary resuscitation, and had his airway secured in the field. Upon arrival to the ED, the patient was noted to be alert and responsive. The patient had received no advanced care life support medications in the field or upon arrival to ED.

**Figure 1:**
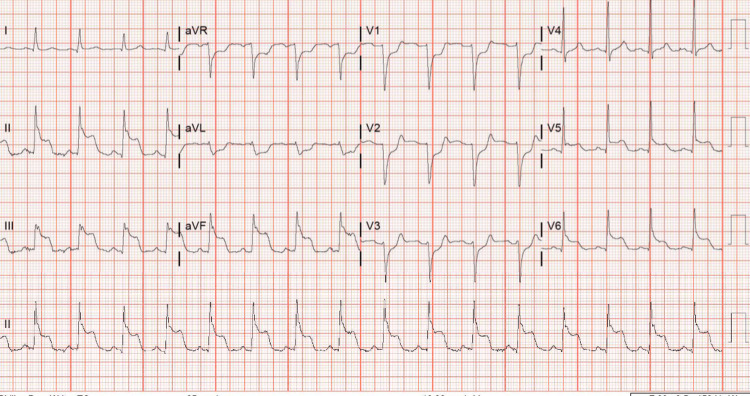
Electrocardiogram showing ST-segment elevation in the leads II, III, avF, V5, and V6 with reciprocal depression in anteroseptal leads

**Figure 2:**
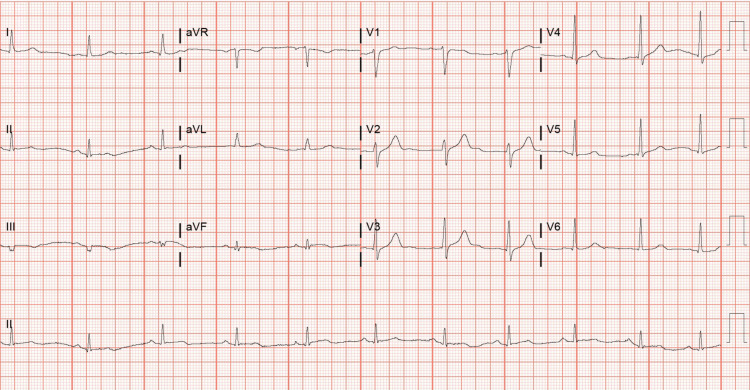
Electrocardiogram showing resolution of both ST-segment elevation in inferior leads and reciprocal depression in anteroseptal leads following cardiac catheterization

Initial triage revealed chest trauma with bilateral pulmonary contusions and sternal fracture with retrosternal hematoma. While in the emergency room, the patient was noted to develop polymorphic ventricular tachycardia, which was resistant to treatment with magnesium sulfate and amiodarone. The subsequent 12-lead electrocardiogram showed > 5-mm ST-segment elevation in the leads II, III, and aVF, 3-mm ST-segment elevation in leads V5 and V6, and reciprocal depression in the anteroseptal leads (Figure 1). High-sensitivity troponin at the time was 142 ng/L. STEMI protocol was activated, and the patient underwent emergent cardiac catheterization. Coronary angiogram revealed 95% stenosis of the middle segment of right coronary artery (RCA) and a drug-eluting stent was placed following pre-dilation of the culprit vessel. Electrocardiogram following catheterization showed resolution of both ST-segment elevation in inferior leads and reciprocal depression in anteroseptal leads (Figure 2). Post-procedure, the patient was started on DAPT consisting of aspirin 81 mg daily and ticagrelor 90 mg twice daily following an initial loading dose of ticagrelor 180 mg. Lisinopril 20 mg daily, atorvastatin 80 mg daily, and metoprolol tartrate 12.5 mg twice daily were also started after PCI. Transthoracic echocardiogram (TTE) following the PCI reported a left ventricular ejection fraction of 56%, with normal global and regional systolic left ventricular function except a moderate hypokinesis of the apical septum.

**Figure 3:**
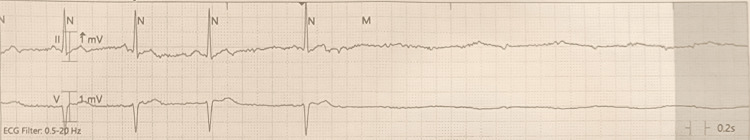
Patient’s telemetry strip showing sinus pause

**Figure 4:**
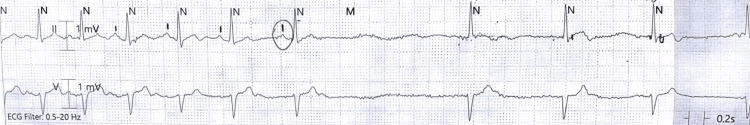
Patient’s telemetry strip showing sinus pause followed by junctional rhythm

The day following percutaneous coronary angioplasty and initiation of ticagrelor, telemetry review revealed multiple sinus pauses lasting around 5-10 seconds each. The longest pause was approximately 12 seconds (Figure 3). After one of the pauses, the patient would enter a junctional rhythm (Figure 4). The patient was awake and asymptomatic during these episodes. Based on discussions between general cardiology, interventional cardiology, and electrophysiology, ticagrelor was suspected to be responsible for the episodes of bradycardia and sinus pauses. The patient’s antiplatelet therapy was switched from ticagrelor to clopidogrel. Metoprolol tartrate was held initially but resumed after 48 hours. Following the transition to clopidogrel, the patient had no further episodes of bradycardia or sinus pauses.

## Discussion

Adenosine diphosphate activation of P2Y12 receptors results in platelet aggregation via glycoprotein IIb/IIIa. The P2Y12 inhibitor drug class impedes platelet aggregation, making it an effective drug after stent placement following ACS [8]. Although this is ticagrelor’s primary mechanism of action, it also blocks hENT1 transporter, thereby increasing serum adenosine levels [9]. Additionally, serum adenosine levels are also known to rise during ischemia. Adenosine slows conduction through the atrial and nodal tissues by opening acetylcholine-sensitive potassium channels and blocking calcium influx [10]. This paper seeks to make physicians aware that ticagrelor use after ACS with PCI can potentially result in significant sinus pauses. This is the result of the independent yet additive elevations of serum adenosine levels by both ischemia and ticagrelor. Wallentin et al. conducted a multicenter, double-blind, placebo-controlled randomized clinical trial investigating the efficacy of ticagrelor versus clopidogrel in the prevention of cardiovascular outcomes. The PLATO trial showed that ticagrelor significantly decreased death from vascular causes, myocardial infarction, or stroke as compared to clopidogrel without a significant increase in the risk of bleeding. While the researchers did observe an increased incidence of ventricular pauses in this group, pauses were noted to be asymptomatic and only significant in the first week of drug initiation [11].

In addition to ischemia, however, there are many components to this patient’s presentation that could have contributed to his sinus pauses, including heroin abuse, administration of amiodarone, beta-blockade, and blunt cardiac injury. Although heroin is known to cause various arrhythmias because of inhibition of sympathetic activity and augmentation of parasympathetic activity, it has a half-life of 2-6 minutes. Heroin is primarily metabolized in the liver into morphine and 6-acetylmorphine, both of which have half-lives of less than 6 hours in those with normal liver function such as outpatients [12,13]. This was not likely responsible for the sinus pauses noted in our patient, as his first pause occurred approximately 44 hours after admission. The onset of action of a single dose of IV amiodarone is 1 hour, and it remains in one’s system for up to 47 days [14,15]. Therefore, if amiodarone was the causative agent, the pauses should have presented sooner in the hospital course and continued to occur throughout admission. Although the patient started having sinus pauses after metoprolol tartrate was initiated and stopped after the drug was discontinued, it is reasonable to assume that his sinus pauses were not a result of the beta blocker as he did not have sinus pauses upon resuming it the following day. Even though the echocardiogram was not indicative of trauma, it is reasonable to assume that the patient had a blunt cardiac injury given that he had bilateral pulmonary contusions as well as a displaced sternal fracture. Although blunt trauma could have led to these arrhythmias, it is unlikely that it would have presented >44 hours after his injury. Lastly, both ischemia and cardiac catheterization with stent placement could have played a role in the patient’s sinus pauses as his RCA was significantly occluded. The RCA supplies the SA node; thus, occlusion of the RCA can lead to sinus arrhythmias [16]. However, it has been shown that these arrythmias usually occur within 24 hours and only with proximal vessel occlusion [17]. Given that the patient underwent PCI shortly after ECG changes, the echocardiogram following the cardiac catheterization revealed no wall motion abnormalities along RCA distribution, and our patient’s occlusion was mid-segment not proximal, it is of low probability that ischemic changes to his SA node caused the witnessed sinus pauses. A reperfusion-induced arrhythmia is another possibility; however, these typically occur within the first 6 hours after thrombolysis, therefore making it unlikely to have been responsible [18]. For these reasons, it is believed that the administration of ticagrelor in the presence of ischemia likely increased adenosine levels to a concentration high enough to result in the documented sinus pauses in this case.

A sub-study of the PLATO trial found that compared to patients on clopidogrel, those on ticagrelor experienced a statistically significant increase in sinus pauses [5]. As with our patient, these occurred more frequently in the acute phase of ACS; however, the existing study did not find any clinically significant consequences to these sinus pauses. Although our patient was asymptomatic during these episodes, the 12-second duration of our patient’s pause was felt to be well outside the generally acceptable limit of what could be safely and prudently tolerated.

## Conclusions

It is well documented that ticagrelor can lead to bradyarrhythmias such as sinus pauses. Although these pauses are thought to have little to no clinical significance, clinicians should be aware of this potential side effect in order to best manage their patients. If providers feel that ticagrelor is contributing to a patient's arrhythmia, they should promptly transition to an alternative P2Y12 inhibitor.
